# Public Schools

**Published:** 1867-06

**Authors:** 


### PUBLIC SCHOOLS.

A school committee from Boston having visited officially the public schools of the larger cities of the country, say of those in New York City ;

"Under the administration of the system as carried out by the Board of Education a degree of order, precision and energy of action has been attained which has carried, and, if persevered in, must continue to carry forward the great work of popular education in the City of New York, with a steady and strong progress, both in the broadness of its diffusion and the excellence of its character."

Of all the principals of these schools, none deserve a higher credit than Mrs. Hall of the Twelfth St., and Hunter of the Boys' school in 13th St ; their long connection with the schools, their large experience, and that just knowledge of child nature would make their places very difficult of being filled, if indeed it were possible. Not long ago, Mr. Hunter was elected to a higher and more lucrative position ; the little fellows under his care were greatly distressed ; one of them on hearing it said very mournfully, "I guess all the boys will leave if Mr. Hunter does." "Why, my child?" "Because they like him so much." "But why do they like him?" "Why he encourages us." There is philosophy for school management from the equator to the poles ; and for parents as well. "He encourages us." Philosophy for masters, for officers, for all who are in authority. He encourages us. And to show what a strong chain "encouragement" forges, the boys of this same school, averaging a dozen years or less, when they heard, during the war that their beloved teacher was drafted, immediately set about collecting their pennies, their half dimes and other loose change, and before he knew anything about it, they had handed over three hundred dollars to pay for a substitute. When the masses have received the benefits of a public school education, and when the heart has been so influenced by the claims of conscience and of duty, as to make men willing to deny themselves all bodily indulgences, then may we expect wise lives on the part of all, leading to high health, great efficiency and general usefulness ; for when the brain and the heart are properly educated, there will follow wisdom, temperance, a long and healthful life, and universal thrift.

